# Admission and outcomes of COVID-19 among chronic obstructive pulmonary diseases patients in Africa: protocol for a systematic review and meta-analysis

**DOI:** 10.1093/inthealth/ihae062

**Published:** 2024-10-03

**Authors:** Guesh Mebrahtom, Abrha Hailay, Woldu Aberhe, Kidane Zereabruk, Teklehaimanot Gereziher Haile, Degena Bahrey Tadesse

**Affiliations:** Department of Adult Health Nursing, School of Nursing, College of Health Science, Aksum University, Aksum, Ethiopia; Department of Adult Health Nursing, School of Nursing, College of Health Science, Aksum University, Aksum, Ethiopia; Department of Adult Health Nursing, School of Nursing, College of Health Science, Aksum University, Aksum, Ethiopia; Department of Adult Health Nursing, School of Nursing, College of Health Science, Aksum University, Aksum, Ethiopia; Department of Maternity and Neonatal Nursing, School of Nursing, College of Health Science, Aksum University, Aksum, Ethiopia; Department of Adult Health Nursing, School of Nursing, College of Health Science, Aksum University, Aksum, Ethiopia

**Keywords:** admission, Africa, chronic obstructive pulmonary disease, COVID-19, outcome

## Abstract

When the coronavirus case was originally reported in Wuhan, China, in December 2019, it quickly spread throughout the world and became a global public health problem. Evidence of the admission and outcomes of coronavirus disease among patients with chronic obstructive pulmonary disease (COPD) has not been reported in Africa. Consequently, this research protocol uses a systematic review and meta-analysis of the admission and outcomes of COVID-19 in patients with COPD in Africa. All observational studies published in the English language and reporting on the prevalence, admission and outcomes of COVID-19 among patients with COPD in Africa will be included. A search strategy will be implemented using electronic databases and the Preferred Reporting Items for Systematic Reviews and Meta-Analysis Protocol recommendations. The findings of this review will be reported to health program designers, decision-makers and healthcare providers.

## Introduction

The emergence in Wuhan City, Hubei Province, China of a novel pneumonia of unknown origin on 31 December 2019 was the start of an outbreak that would later be declared a pandemic by the World Health Organization (WHO).^1^ COVID-19 is caused by a novel strain of coronavirus, the severe acute respiratory syndrome coronavirus 2 (SARS-CoV-2).^2^ This virus belongs to the family of single-stranded RNA viruses, some of which have been previously described to be responsible for severe acute respiratory syndrome (SARS) and Middle East respiratory syndrome (MERS).^3,4^ Medical manifestations of COVID-19 range from asymptomatic illness to flu-like illness, including high morbidity and multi-organ failure mortality.^5^ Most patients diagnosed with COVID-19 had moderate symptoms, including throat pain, dry cough and fever. Many of them spontaneously resolved. Several fatal complications have been reported by others, such as septic shock, severe pneumonia and organ failure.^6^

‘Globally, as of 5:43pm CEST, 17 May 2023, there have been 766 440 796 confirmed cases of COVID-19, including 6 932 591 deaths, reported to WHO. As of 9 May 2023, a total of 13 350 530 518 vaccine doses have been administered. In Africa, 9 530 213 total number of confirmed cases, including 173 365 number of deaths, reported to WHO’.

According to numerous studies, the clinical characteristics of COVID-19 infection are group-based and have shown a higher risk of developing pneumonia as a serious form of infection in the elderly and those with chronic comorbidities, especially systemic arterial hypertension, diabetes mellitus and immunosuppression.^7–9^ Chronic obstructive pulmonary disease (COPD) is a prevalent, chronic and preventable pulmonary disorder associated with airflow failure. COPD is a complicated disease associated with airway and/or alveoli defects, primarily caused by long-term exposure to noxious gases and particulates.^10^ COPD exacerbations are a major event in the natural history of the disease, associated with worsening of symptoms and often resulting in hospitalization and poor prognosis.^11^ Various factors have been described to contribute to acute worsening of COPD, however, viral infection remains the main trigger, including seasonal coronaviruses.^12–14^ The prevalence of COPD among COVID-19 patients was 2%.^15^ Also, patients with pre-existing COPD infected with COVID-19 are more likely to have a higher risk of mortality than COPD-free patients.^15,16^ There is, however, no combined result of the admission and outcomes of COVID-19 among COPD patients in Africa. Consequently, this research protocol uses a systematic review and meta-analysis of the admission and outcomes of COVID-19 in patients with COPD in Africa.

## Methods

### Study protocol and systematic review registration

This research protocol uses a systematic review and meta-analysis of the admission and outcomes of COVID-19 among patients with COPD in Africa. This review will be reported according to the Preferred Reporting Items for Systematic reviews and Meta-Analysis Protocol (PRISMA-P) guidelines.^17^ This review is registered in the PROSPERO International Prospective Register of Systematic Reviews with the registration number CRD42020215754 (https://www.crd.york.ac.uk/prospero/#recordDetails).

### Study design and setting

A systematic review and meta-analysis that included all studies that report the prevalence, admission and outcomes of COVID-19 among patients with COPD and were conducted in hospitals or intensive care units will be employed.

### Search strategy and information sources

A comprehensive search strategy will be implemented using electronic databases (PubMed/MEDLINE, Embase, Google Scholar, Web of Science, Cochrane Library, Africa-Wide Information, Africa Index Medicus, Scopus, Africa Journal Online, World Health Organization (WHO) Afro Library and Cochrane (see Table 1). The search will be carried out either individually or with the following keywords in combination: admission, chronic obstructive pulmonary disease, chronic obstructive lung disease, chronic obstructive airway disease, COVID-19, outcome and prevalence. Search terms used will include ‘Wuhan coronavirus’ OR ‘COVID-19’ OR’ novel coronavirus‘ OR ’2019-nCoV‘ OR ’Coronavirus outbreak‘ OR ’SARS-CoV-2‘ OR ’SARS2‘ OR ’Severe acute respiratory syndrome coronavirus 2‘ OR’ Outcome. Other searching terms are ‘death’ OR ‘mortality’ OR ‘morbidity’ OR ‘prevalence’ OR ‘incidence’ OR ‘chronic obstructive pulmonary disease’, ‘chronic obstructive airway disease’, ‘chronic obstructive lung disease’ and ‘complication of COVID-19’.

**Table 1.** Search strategy and information source

Databases	Articles found, *n*	Articles included, *n*	Articles excluded, *n*	Reason for exclusion
Google Scholar	?	?	?	Irrelevant or duplicate
PubMed/MEDLINE	?	?	?	Irrelevant or duplicate
EMBASE	?	?	?	Irrelevant or duplicate
HINARI	?	?	?	Irrelevant or duplicate
Cochrane Library	?	?	?	Irrelevant or duplicate
WHO COVID-19 database	?	?	?	Irrelevant or duplicate
Africa-Wide Information	?	?	?	Irrelevant or duplicate
Web of Science	?	?	?	Irrelevant or duplicate
Scopus	?	?	?	Irrelevant or duplicate
Africa Index Medicus	?	?	?	Irrelevant or duplicate
Africa Journals Online	?	?	?	Irrelevant or duplicate
Unpublished thesis, manuscript and report from the WHO and CDC	?	?	?	Irrelevant or duplicate

### Data extraction and quality assessment

The data extraction will be performed by two independent reviewers using a predefined and structured method of data collection. Two assessors (GM and WA) will use the predefined standardized extraction form from the included studies to autonomously extract data. The extraction of data includes first author, year and/or month of publication, country and/or region, signs and symptoms, outcomes (complications, recovery and death), diagnostic criteria, comorbidity, COVID-19, area of study, prevalence and/or incidence, study characteristics such as design of the study and response rate. Disagreements will be settled through discussion and after mutual consensus between the two reviewers. When there is missing information, the corresponding author of the study will be contacted to provide the necessary details. Up to three e-mails will be sent to the corresponding author for additional information before considering exclusion. For studies that occur in multiple publications, we will use the most recent, comprehensive or with the greatest sample size. For surveys that appear in a single article with several surveys done at various times, each survey is treated as an independent study.

### Criteria for considering studies for the review

#### Inclusion criteria

- Study design: All observational research will be included, including cross-sectional studies, cohort studies, case–control studies and baseline findings from randomized controlled trials conducted in Africa among COVID-19 patients with pre-existing COPD.
- Participants/population: All pre-existing COPD patients living in Africa who have been laboratory-confirmed and/or clinically diagnosed with COVID-19.
- Settings/area: Studies conducted in hospitals.
- Intervention(s), exposure(s): COPD with an outbreak of COVID-19. We therefore want to examine the admission and outcomes of COVID-19 in COPD patients.
- Outcomes: COVID-19 morbidity and clinical outcomes, including infection and prevalence rates, rates of recovery, complications and mortality, among individuals with pre-existing COPD.
- Language: Only published articles in the English language will be included in this study because of feasibility.
- Method of diagnosis: There will be no limitations on diagnostic procedures, but a subgroup analysis focused on diagnostic tools will be carried out. WHO interim guidance and/or any diagnostic requirements suggested by the WHO shall be considered ‘WHO interim guidance for 2019-nCoV-related laboratory biosafety’^18^ (see Table 2).

**Table 2.** Laboratory examination of coronavirus disease in suspected human cases: interim guidance

Test	Type of sample	Timing
Nucleic acid amplification tests (NAATs)	Upper respiratory specimens: nasopharyngeal and oropharyngeal swab, nasopharyngeal wash/nasopharyngeal aspirateLower respiratory specimens: sputum, endotracheal; aspirate or bronchoalveolar lavage are preferred for patients with severe respiratory disease	Upon introduction. May be repeated sampling to monitor clearance. Additional work is required to assess if repeated sampling is efficient and accurate
Serology and other	blood and stool are also responsible for the coronaviruses (COVID-19)	Paired samples are needed to confirm with the original sample obtained during the first week of the disease and the second one preferably obtained after 2–4 weeks. (There has to be an ideal timing for convalescent samples.)

#### Exclusion criteria

The following studies will be excluded from consideration: case reports, case series studies, studies lacking the pertinent information required to calculate the morbidity and clinical outcomes of COVID-19 among COPD patients in Africa, duplicated articles, protocol articles and review articles.

### Quality assessment and risk of bias in individual studies

The risk of bias and validity of included studies will be assessed using a method developed by Hoy et al.^19^ for prevalence studies. The tool includes 11 items: items 1–4 evaluate the external validity, items 5–10 evaluate the internal validity and item 11 provides the reviewer with a summary of the overall risk based on the responses of the 10 items above that are scored 1 if yes and 0 if no. Studies are rated as low risk (<3), moderate risk (4–6) or high risk of bias (7–9). This activity will be done by two reviewers and disagreements will be settled by discussion and, if needed, by arbitration involving a third author. Furthermore, appropriate sampling methods, consistent data collection methods and procedures, documented quality control methods and a representative sample size will be regarded as indicators of the quality of the research. High-quality studies are studies that have revealed all the above points.

### Data management

A structure was established a priori, based on the inclusion and exclusion criteria, to guide the screening and selection process. Until data extraction proceeds, the tool will be piloted and updated. Next, the search results will be submitted to the EndNote software (Clarivate, London, UK) to remove duplicates. Rayyan (https://www.rayyan.ai/), a smartphone and a web-based software framework, will be used on the remaining papers, enabling cooperation between reviewers involved in the screening and selection of studies.^20^

### Data analysis and presentation of results

During the analysis of the results, R and R Studio version 3.5.3 (Foundation for Statistical Computing, Vienna, Austria) will be used. All analysis will be performed using a metaprop routine for Windows using R version 3.5.3.^21^ Results with corresponding 95% confidence intervals will be reported as proportions. To reflect the pooled outcomes of COVID-19 and the degree of statistical heterogeneity between studies, forest plots will be drawn. Using the χ^2^ measure, statistical heterogeneity will be evaluated and quantified using the *I*^2^ statistics equation, with values of 25%, 50% and 75% reflecting low, medium and high heterogeneity, respectively.^22^ If there is heterogeneity between studies, we will use a meta-analysis of random effects^23^ to estimate the aggregate pooled prevalence, admission and outcomes of COVID-19 among COPD patients in Africa. The funnel plot test and Egger's test methods will be used to determine potential publication bias.^24^ On the Egger scale, a p-value <0.10 is considered statistically significant for bias in reporting. A subgroup analysis will be conducted by the study country or geographic location and based on clinical outcomes such as mortality, recovery rate and disease severity. A sensitivity analysis will be conducted to determine if the results of an individual study significantly influenced the combined outcome. The systematic review's overall evidence quality will be evaluated based on the Grading of Recommendations Assessment, Development, and Evaluation guidelines, considering factors such as imprecision, bias risk, publication bias and heterogeneity. Following specific evaluation criteria, the evidence quality will be categorized as high, moderate, low or very low.^25^

### Study selection and data collection process

The data will be extracted using a standardized data extraction technique. Two assessors (GM and WA) will use the predefined standardized extraction form to autonomously extract data. Full texts for the qualifying titles and/or abstracts, including those where uncertainty exists, will be collected for further consideration of inclusion in the study. The agreement between the study reviewers will be calculated using the statistics from Cohen's λ. Disagreements will be settled by discussion and, if necessary, arbitration by a third reviewer (AH). Reasons for exclusion will be noted.

Where there are missing data, authors will be contacted for additional information to ensure study eligibility. Up to three e-mails will be sent to the corresponding author to request additional information before the research will be excluded. For studies that appear in more than one published paper, we will consider the newest, most comprehensive and largest sample size. For surveys appearing in one article with several surveys performed at various time points, we will treat each survey as a separate report. The data extracted will include first author, month of publishing, country and/or region, signs and symptoms, complications, diagnostic criteria, comorbidity, COVID-19, area of study, prevalence and/or incidence, study characteristics such as the study design and response rate.

### Outcomes and prioritization

The primary outcome is the admission and outcomes of COVID-19 among COPD patients in Africa.

### Data synthesis

Crude numerators and denominators from individual studies will be used to calculate the study-specific outcomes of COVID-19 among COPD patients. A meta-analysis will be conducted on variables that are consistent across the included research. Since there will be heterogeneity among the studies, the pooled admission and outcomes of COVID-19 in Africa will be determined by a random effects model. A subgroup analysis will summarize the African geographic regions, diagnostic tools and, based on their ethnic context, where the research was carried out.

## Discussion

**Figure 1.**
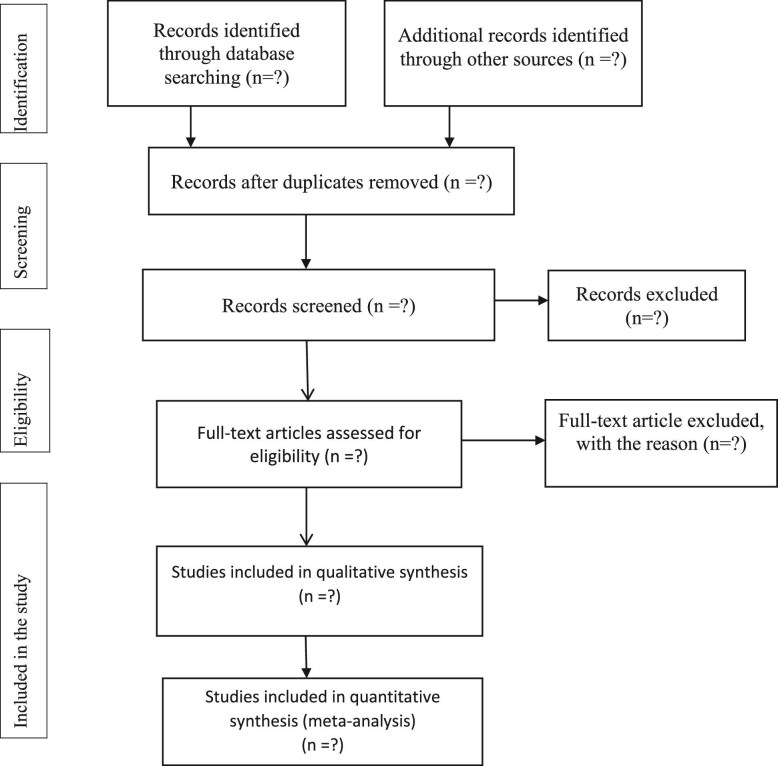
Selection of articles for systemic review and meta-analysis of burden, admission and outcomes of COVID-19 among COPD patients in Africa.

The aim of this systematic review and meta-analysis is to assess the admission and outcomes of COVID-19 among COPD patients across Africa. This review will be done on the basis of the PRISMA-P guidelines and the PRISMA flow diagram and will be used to document the different phases of the review process^17^ (see Figure 1). This study will focus on analysing COVID-19 morbidity and clinical outcomes such as infection and prevalence rates, recovery rates, complications and mortality among individuals with underlying COPD based on existing literature. It will encompass an investigation into symptoms, laboratory and imaging findings (including chest X-rays, CT scans, C-reactive protein levels and complete blood counts) and spirometry data concerning COPD outcomes (recovery, complications and mortality) in relation to COVID-19. The outcomes of this review will provide valuable insights to health program planners, decision-makers and healthcare professionals, facilitating the delivery of better-quality healthcare services, emphasizing critical issues and enhancing clinical practices in Africa. The findings from this research will be disseminated through conferences, peer-reviewed journals and social media platforms.

## Conclusions

The admission and outcomes of COVID-19 among COPD patients in Africa will be quantified by this systematic examination and meta-analysis.

## Data Availability

The datasets used and/or analysed during the analysis are available upon request to the corresponding author.

## References

[1] World Health Organization . Coronavirus disease (COVID-2019) situation reports. Available from: https://www.WHO.Int/docs/default-source/coronavirus/situationreports/20200221-sitrep-32-covid

[2] Jiang S, Shi Z, Shu Y et al. A distinct name is needed for the new coronavirus Lancet. 2020;395(10228):949. 10.1016/S0140-6736(20)30419-0 PMC7124603 32087125

[3] Robinson KS, Aw R. The commonalities in bacterial effector inhibition of apoptosis. Trends Microbiol. 2016;24(8):665–80. 27117049 10.1016/j.tim.2016.04.002

[4] Zhu N, Zhang D, Wang W et al. A novel coronavirus from patients with pneumonia in China, 2019 N Engl J Med. 2020;382(8):727–33. 31978945 10.1056/NEJMoa2001017 PMC7092803

[5] Chen N, Zhou M, Dong X et al. Epidemiological and clinical characteristics of 99 cases of 2019 novel coronavirus pneumonia in Wuhan, China: a descriptive study. Lancet. 2020;395(10223):507–13. 32007143 10.1016/S0140-6736(20)30211-7 PMC7135076

[6] Zaim S, Chong JH, Sankaranarayanan V et al. COVID-19 and multi-organ response Curr Probl Cardiol. 2020;45(8):100618. 32439197 10.1016/j.cpcardiol.2020.100618 PMC7187881

[7] Mohammed M, Muhammad S, Mohammed FZ et al. Risk factors associated with mortality among patients with novel coronavirus disease (COVID-19) in Africa J Rac Ethnic Health Dispar. 2021;8(5):1267–72. 10.1007/s40615-020-00888-3 PMC7553376 33051749

[8] Du Y, Tu L, Zhu P et al. Clinical features of 85 fatal cases of COVID-19 from Wuhan. A retrospective observational study. Am J Respir Crit Care Med. 2020;201(11):1372–9. 32242738 10.1164/rccm.202003-0543OC PMC7258652

[9] Chen H, Guo J, Wang C et al. Clinical characteristics and intrauterine vertical transmission potential of COVID-19 infection in nine pregnant women: a retrospective review of medical records. Lancet. 2020;395(10226):809–15. 32151335 10.1016/S0140-6736(20)30360-3 PMC7159281

[10] Savran O, Godtfredsen N, Sørensen T et al. COPD patients prescribed inhaled corticosteroid in primary care–time for re-assessment based on exacerbation rate and blood eosinophils? Respir Res. 2021;22(1):54. 33579297 10.1186/s12931-021-01651-w PMC7881579

[11] Seemungal TAR, Donaldson GC, Paul EA et al. Effect of exacerbation on quality of life in patients with chronic obstructive pulmonary disease. Am J Respir Crit Care Med. 1998;157(5):1418–22. 9603117 10.1164/ajrccm.157.5.9709032

[12] White AJ, Gompertz S, Stockley R. Chronic obstructive pulmonary disease • 6: the aetiology of exacerbations of chronic obstructive pulmonary disease. Thorax. 2003;58(1):73–80. 12511727 10.1136/thorax.58.1.73 PMC1746462

[13] Harausz EP, Garcia-Prats AJ, Seddon JA et al. New and repurposed drugs for pediatric multidrug-resistant tuberculosis. Practice-based recommendations Am J Respir Crit Care Med. 2017;195(10):1300–10. 27854508 10.1164/rccm.201606-1227CI

[14] Wedzicha JA, Seemungal TA. COPD exacerbations: defining their cause and prevention. Lancet. 2007;370(9589):786–96. 17765528 10.1016/S0140-6736(07)61382-8 PMC7134993

[15] Alqahtani JS, Oyelade T, Aldhahir AM et al. Prevalence, severity and mortality associated with COPD and smoking in patients with COVID-19: a rapid systematic review and meta-analysis. PLoS One. 2020;15(5):e0233147. 32392262 10.1371/journal.pone.0233147 PMC7213702

[16] Hu W, Dong M, Xiong M et al. Clinical courses and outcomes of patients with chronic obstructive pulmonary disease during the COVID-19 epidemic in Hubei, China. Int J Chron Obstruct Pulmon Dis. 2020;15:2237–48. 33061341 10.2147/COPD.S265004 PMC7520151

[17] Moher D, Shamseer L, Clarke M et al. Preferred reporting items for systematic review and meta-analysis protocols (PRISMA-P) 2015 statement Syst Rev. 2015;4(1):1. 25554246 10.1186/2046-4053-4-1 PMC4320440

[18] World Health Organization . Laboratory testing for coronavirus disease 2019 (COVID-19) in suspected human cases: interim guidance, 2 March 2020. Geneva: World Health Organization. Available from: https://iris.who.int/handle/10665/331329

[19] Hoy D, Brooks P, Woolf A et al. Assessing risk of bias in prevalence studies: modification of an existing tool and evidence of interrater agreement J Clin Epidemiol. 2012;65(9):934–9. 22742910 10.1016/j.jclinepi.2011.11.014

[20] Ouzzani M, Hammady H, Fedorowicz Z et al. Rayyan—a web and mobile app for systematic reviews Syst Rev. 2016;5(1):210. 27919275 10.1186/s13643-016-0384-4 PMC5139140

[21] Nyaga VN, Arbyn M, Aerts M. Metaprop: a Stata command to perform meta-analysis of binomial data. Arch Public Health 2014;72(1):39. 25810908 10.1186/2049-3258-72-39 PMC4373114

[22] Higgins JP, Whitehead A, Turner RM et al. Measuring inconsistency in meta-analyses BMJ. 2003;327(7414):557–60. 12958120 10.1136/bmj.327.7414.557 PMC192859

[23] DerSimonian R, Laird N. Meta-analysis in clinical trials. Control Clin Trials. 1986;7(3):177–88. 3802833 10.1016/0197-2456(86)90046-2

[24] Egger M, Smith GD, Schneider M et al. Bias in meta-analysis detected by a simple, graphical test BMJ. 1997;315(7109):629–34. 9310563 10.1136/bmj.315.7109.629 PMC2127453

[25] Berkman ND, Lohr KN, Ansari MT et al. Grading the strength of a body of evidence when assessing health care interventions: an EPC update J Clin Epidemiol. 2015;68(11):1312–24. 25721570 10.1016/j.jclinepi.2014.11.023

